# An evaluation of four modes of low-dose anticoagulation during intermittent haemodialysis

**DOI:** 10.1007/s00228-017-2389-x

**Published:** 2017-12-02

**Authors:** Malin S. E. Skagerlind, Bernd G. Stegmayr

**Affiliations:** 1 Department of Public Health and Clinical Medicine, University of Umea, Umea, Sweden; 2 Department of Nephrology, Centre of Medicine, University Hospital of Umea, 90185 Umea, Sweden

**Keywords:** Haemodialysis, Haemorrhage, Priming, Anticoagulation

## Abstract

**Introduction:**

Intensive care participants that need dialysis frequently suffer from increased risk of bleeding. Standard intermittent haemodialysis (SHD) includes anticoagulation to avoid clotting of the dialysis system. The aim of this study was to clarify which of four different low-dose anticoagulant modes was preferable in reducing the exposure to i.v. unfractionated heparin (heparin) and maintaining patency of the dialysis circuit.

**Methods:**

Twenty-three patients on SHD were included to perform haemodialysis with four modes of low-dose anticoagulation. For comparative analyses, patients served as their own control. Haemodialysis with a single bolus of tinzaparin at the start was compared to haemodialysis initiated without i.v. heparin but priming with (1) heparin in saline (H), (2) heparin and albumin in saline (HA), (3) heparin and albumin in combination with a citrate-containing dialysate (HAC), (4) saline and usinga heparin-coated filters (Evodial®). The priming fluid was discarded before dialysis started. Blood samples were collected at 0, 30 and 180 min during haemodialysis. Smaller bolus doses of heparin (500 Units/dose) were allowed during the modes to avoid interruption by clotting.

**Findings:**

The mean activated partial thromboplastin (APTT) time as well as the doses of anticoagulation administered was highest with SHD and least with HAC and Evodial®. Mode H versus SHD had the highest rate of prematurely interrupted dialyses (33%, *p* = 0.008). The urea reduction rate was less with Evodial® vs. SHD (*p* < 0.01). One hypersensitivity reaction occurred with Evodial®. Changes in blood cell concentrations and triglycerides differed between the modes.

**Discussion:**

If intermittent haemodialysis is necessary in patients at risk of bleeding, anticoagulation using HAC and Evodial® appeared most preferable with least administration of heparin, lowest APTT increase and lowest risk for prematurely clotted dialyzers in contrast to the least plausible H mode.

**Electronic supplementary material:**

The online version of this article (10.1007/s00228-017-2389-x) contains supplementary material, which is available to authorized users.

## Background

Standard intermittent haemodialysis (SHD) includes anticoagulation with unfractionated heparin (heparin) or low molecular weight heparin (LMWH) to avoid clotting of the extracorporeal circuit of the dialysis system [1].

In patients at risk of bleeding, while needing haemodialysis, one option is to use intravenous regional citrate infusion. This technique is so far only commercially available for continuous veno-venous haemodialysis (CVVHD) used in intensive care units and needs narrow clinical and laboratory surveillance. Except for a few centres with developed methods of narrow surveillance [4, 6, 10, 11], regional citrate anticoagulation (RCA) therefore is considered unsuitable for intermittent haemodialysis due to the need of extensive surveillance, to avoid risk of hypo- or hypercalcaemia. In untrained hands, RCA has been recommended to be limited to intensive care [8]. Therefore, other options may be considered for intermittent haemodialysis in patients at risk of bleeding.

Another way to restrict anticoagulation during intermittent haemodialysisis is by using saline flushes, heparin-coated dialyzers [2–9], dialysis fluid containing citrate[12, 13]or by combining heparin-coated dialyzers with citrate dialysate [14]. However, these methods may end up in frequent clotting (50% interrupted treatments) [15]. A pharmacological heparin coating of the dialyzer and the extracorporeal circuit, without using any heparin at the start, is another option. Such method is the manual priming by perfusion of the extracorporeal circuit with a combination of heparin and albumin that is discarded before intermittent haemodialysis [16, 17]. A prior in vitro study indicated that priming the extracorporeal circuit with only either saline or an albumin solution caused a greater risk for clotting in comparison to priming with heparin in saline or heparin and albumin in saline [18]. Saline flushes can cause fluid retention while regional citrate anticoagulation needs careful surveillance especially of ionized Ca^2+^ [12, 19]. A clotting of the extracorporeal circuit causes interrupted haemodialysis but also a blood loss up to 300 ml. Still, there is no golden standard for anticoagulation during intermittent haemodialysis in participants at bleeding risk [8].

The aim of this study was to clarify to what extent four different low-dose anticoagulant modes, versus standard haemodialysis, could reduce the administration of heparin while enabling dialysis.

## Materials and methods

Participants on chronic intermittent haemodialysis (*n* = 23, 16 male) in a stable condition were included. The participants were consecutively informed and written consent to participate was obtained. Excluded were participants with a weight gain of more than 3 L between dialyses, access problems, acute infections or dementia. The reasons for end-stage kidney disease and intermittent haemodialysis were diabetic nephropathy (*n* = 6), glomerulonephritis (*n* = 5), nephrosclerosis (*n* = 5), polycystic kidney disease (*n* = 4) and interstitial nephritis (*n* = 3). Nine of the participants had diabetes mellitus. Included in daily medications were antiplatelets (acetylsalicylic acid: *n* = 14, clopidogrel: *n* = 1) and anticoagulants (warfarin: *n* = 6, subcutaneous dalteparin: *n* = 1).

At different stages during the study period, five participants dropped out due to change of treatment regime (*n* = 1), impaired health (*n* = 1) and no given reason (*n* = 2). One participant terminated the study after having suffered from a side effect with Evodial®.

Vascular accesses were arterial venous fistula (AV fistula, *n* = 12), central dialysis catheter (*n* = 10) and femoral dialysis catheter (n = 1). The catheter lock solutions used were heparin 5000 Units/ml (*n* = 5), and TauroLock**™**-HEP500 [(cyclo)-taurolidine, citrate 4% and heparin (mucosa 5000 IU/ml)] (Tauro-Implant GmbH, Winsen, Germany) (*n* = 5).

Dialysis devices were Gambro Artis™ with tubing system Artiset™ (Gambro Dasco S.p.A. Modella, Italy), Fresenius 5008 with tubing system Life Line Beta AV-Set ONLINEplus BVM 5008-R (Fresenius Medical Care AG & Co. Bad Homburg, Germany) and Fresenius 4008 with tubing system DiaLine A/V set (F.M. S.p.A., Cigliano, Italy).

The dialyzers used were FX80 (1.8 m^2^, Fresenius Medical Care, AG & Co. Bad Homburg, Germany) for all dialyses except when Evodial® dialyzers (1.6 m^2^, Baxter Gambro, Lund, Sweden) were used.

The dialysates used were Smartbag® 211.25 or 311.25 (Fresenius Medical Care, Bad Homburg, Germany), and contained K^+^ 2.0 or 3.0 mmol/L, Ca^2+^ 1.25 mmol/L, acetate 3.0 mmol/L and glucose 1 g/L. During the heparin-albumin priming and additional citrate in the dialysate (HAC mode) the SelectBag® CX265G Citrate (Gambro Dasco S.p.A. Modela, Italy) dialysate was used, with a final concentration of K^+^ 2.0 mmol/L, Ca^2+^ 1.65 mmol/L, citrate 1.0 mmol/L and glucose 1 g/L.

The study was performed at a single centre and prospective with each patient being its own control (case-control design). Data from a base line standard dialysis of each patient was used as comparison to four other modes, using low-dose anticoagulation, for the same patient (Supplement Table 1). Safety aims were to avoid interrupted dialyses due to total clotting as much as possible. Thereby, nurses were informed to check, i.e. pressures within the extracorporeal system and tendency of progressive clotting of dialyzers and arterial and venous chambers. In addition, the awareness of specific dialyzer would help the nurse to interpret eventual clinical side effects.

**Table 1:** Anticoagulation, APTT, urea reduction rate and blood volume in mean for each treatment mode. Mean ± SD and range (when appropriate) is given for added unfractionated heparin (heparin), activated partial thromboplastin time (APTT), urea reduction rate (URR) and blood volume dialysed per haemodialysis session

	Heparin *n* = 20	HA *n* = 21	HAC *n* = 19	Evodial® *n* = 19	*SHD* *n = 23*
Added heparin*, Units (range)	852 ± 907 (0–2600)	474 ± 713 (0–2500)	274 ± 611 (0–2000)	184 ± 299 (0–1000)	*4413 ± 1838* ^**^ *(2500–9000* ^*^ *)*
Treatments without heparin, %	48	52	52	64	*0*
APTT 30′, sec (range)	37 ± 16 ^a)^ (25–96)	34 ± 7 ^b)^ (26–50)	34 ± 8 ^a)^ (27–56)	31 ± 4 ^a)^ (25–40)	*98 ± 39* *(49–181)*
APTT 180′sec (range)	38 ± 21 (25–110)	30 ± 3 ^c)^ (23–34)	30 ± 4 ^b)^ (24–44)	29 ± 3 ^b)^ (24–35)	*48 ± 15* *(30–85)*
URR 30′, %	25 ± 8 ^e)^	28 ± 6^e,f)^	26 ± 9	22 ± 7 ^c)^	*27 ± 6*
URR 180′, %	57 ± 30^e,f)^	61 ± 9 ^e)^	61 ± 8^a,d,e)^	55 ± 15 ^c)^	*63 ± 7*
Blood volumeprocessed, L (range)	56.9 ± 21.3 (1.5–92.9)	61.5 ± 12 (42.8–82.2)	61.9 ± 18 (0–80.5)	60.9 ± 12 (0–83)	*65.1 ± 11.7* *(43–83.3)*
Treatment duration, min (range)	197 ± 64 (5–249)	217 ± 21 (165–240)	209 ± 54 (179–240)	206 ± 55 (10–240)	*220 ± 23* *(177–241)*

Units used until 180 min of dialysis. If stop appeared due to clotting, the dose heparin used was calculated as the even upper 100-Unit value of 20 × body weight

*Unfractionated heparin in all modes except SHD, **Tinzaparin

*HA* heparin-albumin, *HAC* heparin-albumin and citrate, *SHD* standard haemodialysis

^a)^ *p* < 0.05 vs. SHD

^b)^ *p* < 0.01 vs. SHD

^c)^ *p* = 0.001 vs. SHD

^d)^ *p* < 0.05 vs. Evodial®

^e)^ *p* < 0.01 vs. Evodial®

^f)^ *p* < 0.05 vs. HAC

Before the standard intermittent haemodialysis (SHD) (*n* = 23) the extracorporeal system was flushed through with saline (9 mg/ml) or online dialysis fluid, depending on what kind of machine was used. The participants received their regular dose of tinzaparin (LEO Pharma AB, Malmö) at the start of SHD (Supplement Table 1).

The priming fluids containing heparin were discarded before haemodialysis was initiated during the following four low-dose anticoagulant modes:

- Priming the extracorporeal circuit with Heparin in saline (H)—5000 Units/L in saline (9 mg/ml). This mode was motivated by a previous in vitro study where H priming was significantly better than priming with albumin or saline [18].
- Priming the extracorporeal circuit with a combination of heparin and albumin in saline (HA): 5000 Units/L of heparin and 1 g/L of albumin in saline (9 mg/mL). Priming was done with a pump speed of 80 mL/min to minimize foaming and bubbles.
- Priming of the extracorporeal circuit with HA as above combined with citrate dialysate fluid (HAC).
- Priming the extracorporeal circuit with saline (9 mg/mL) only, and using the Evodial® dialyzer that is pre-coated with heparin from the manufacturer.

The study was performed in two steps. In the first step, a randomization was used to decide on starting intermittent haemodialysis with either SHD, H or HA. In the second step, another randomization decided if haemodialysis was started with either HAC or Evodial® (Supplement Table 1). Nurses that were not connected to the patients or to the study performed the randomization.

Priming fluid was not re-circulated. After the priming, the arterial tube was connected to the participant and the blood pushed the priming fluid into a sterile waste bag. When the blood had reached the end of the venous tube, the venous tube was connected to the participant.

To avoid hypokalaemia during HAC (citrasate dialysate contained 2.0 mmol/L of potassium), participants that usually underwent SHD with a dialysate containing K^+^ 3.0 mmol/L received an additional mixture of 1 g of oral potassium citrate before protocol haemodialyses. Sodium and bicarbonate settings in the machine were kept the same as for SHD during all treatments.

To avoid interruption of haemodialysis due to clotting, additional heparin doses (500 Units/dose) were prescribed to be administered if clotting tendencies arose. Clotting was estimated by visual check of the extracorporeal circuit chambers or a rising venous pressure. If > 20 Units of heparin per kilogram of body weight had to be given, the treatment was considered as interrupted due to the high tendency of clotting (occurred in two haemodialyses in two different participants).

Blood samples were collected from the vascular access before the treatment (0 min) and from a sampling membrane at the inflow (arterial) side of the extracorporeal circuit at 30 and 180 min. From the central dialysis catheters 5 ml of blood was aspirated and discarded from each lumen to remove the catheter lock solution before treatment start. Thereafter, flushing and aspiration were done several times, using a 10-ml syringe with saline before blood samples were collected.

Each participant performed one session of each type of anticoagulation. Such intermittent haemodialysis was performed at the same day of the week at all times. In addition, the blood pump was kept similar, as was the general routine for the participant. Dialysate flow was for all 500 mL/min. Samples for laboratory analyses were performed at the start, 30 and 180 min, to enable comparison of data between participants, even if some had longer dialysis periods. Samples were drawn for measurements of concentrations of creatinine (mmol/L), urea (mmol/L), albumin (g/L), haemoglobin (g/L, Hb), erythrocytes (10E9/L), erythrocyte volume fraction (EVF), platelets (10E12/L), leukocytes (10E9/L), granulocytes (10E9/L), lymphocytes (10E9/L), monocytes (10E9/L), basophils (10E9/L) and eosinophils (10E9/L). Samples were also collected to measure activated partial thromboplastin time (APTT, reference 22–37 s; SP Liquid Hemosil IL on ACL Top 700 LA) as an estimate of the interference of heparin on the intrinsic clotting system and factors V and X [20].Also, tinzaparin administration can be estimated by this method since it also consists of multiple fractions of molecules similar to unfractionated heparin [21].Triglyceride concentration (mmol/L) was measured as an indirect marker for a biological effect of heparin on the release of lipoprotein lipase (LPL) from the endothelial surface [22].

To avoid false effects caused by dilution or haemo-concentration due to priming volume or ultrafiltration, the concentration of cells and triglycerides were adjusted to the change in haemoglobin concentration. The ratios of haemoglobin (Hb) at the start vs. Hb at 30 and at 180 min were used. Adjustments for the effect of the ultrafiltration were made. The participants were allowed to eat sandwiches during the haemodialysis.

Data on subjective or objective side effects induced by the dialysis, dialysis time, participant weight, extent of ultrafiltration needed, blood pressure (BP, mmHg) before and after treatment, speed of blood pump and dialysed blood volume were also collected. Clotting of the dialyzer and chambers were after haemodialysis visually evaluated and noted by the nurses, according to the department’s standard protocol. Dialyzer grades: clean, striped, red all over, or total clotted. Chamber grades: clean, collar around the inside, clot formations, or total clotting. If extra doses of heparin were administered due to progressive clotting during dialysis, this was noted in the protocol. Intravenous medication other than heparin was avoided during the treatments.

### Ethics

The study was approved by the local ethical committee (DNR: 2013-313-31M) and the Swedish Medical Product Agency (EUDRACT number: 2010-024449-65) All procedures performed in the study were in accordance with the ethical standards of the institutional and/or national research committee and with the 1964 Helsinki declaration and its later amendments or comparable ethical standards.

### Statistics

The data was blinded before statistical calculations and analyses were done. Differences between modes were calculated using the paired non-parametric Wilcoxon signed-rank test. Minimal sample size of 19 pairs was based on an expected effect size of 0.7, α error of 0.05 and 1-β error of 0.80 (G*Power, V3.1.9.2 or Windows). For group comparisons, the Mann Whitney, the Student test (for normal distributed data) and ANOVA were used (IBM®, SPSS® Statistics edition 21 and 23). Also for comparisons, Fisher’s test was performed using Epi Info™ (Center of Disease Control, Atlanta). A two-tailed *p* value of less than 0.05 was considered as significant.

## Results

The mean (±SD) baseline data for the whole study group was age 67(±12) years, the duration of the haemodialysis session 221(± 23) min/dialysis session, blood haemoglobin 114(±12) g/L, platelets (x10E9/L) 210(± 71), systolic/diastolic blood pressure before haemodialysis 152/74 mmHg, weight before treatment 84(± 19) kg, blood pump speed 304(± 52) mL/min, the ultrafiltration volume 1138(± 989) ml/haemodialysis session and total blood volume processed 65(± 12) L/haemodialysis.

### Safety issues

The mean APTT from the start was 44 s (± 36, median 32). A higher mean APTT value was noted, before haemodialysis, in participants with a central dialysis catheter compared to those with an AV fistula (61 ± 50 vs. 30 ± 6.7 s, *p* = 0.001). At 30 min, the APTT for the central dialysis catheter group became lower and the difference was no longer significant between groups (*p* = 0.082).

Two participants had spontaneously elevated APTT above the reference value throughout the whole study period. APTT increased at the most by 3 s at 30 min (mode H) in them. These participants were excluded from the comparative APTT analyses given below.

Table 1 shows that APTT at 30 and 180 min was lower for all low-dose modes compared to SHD (*p* < 0.05). All low-dose modes, except the H mode, had a mean APTT within the normal range and a lower APTT at 180 min compared to APTT at 30 min.

APTT did *not* differ between dialyses when heparin was *not* given vs. those when bolus doses of heparin were given (mean values at start: 49 ± 40 s vs. 40 ± 36; at 30 min: 38 ± 12 vs. 37 ± 6; at 180 min: 32 ± 7 vs. 33 ± 5 s).

Aside from the SHD dialyses, APTT was above the reference value (37 s) at 30 min and at 180 min, respectively, during H mode in: 8 dialyses and in 4 dialyses, during HA mode in: 6 dialyses and in 0 dialyses, during HAC mode in: 3 dialyses and in 1 dialysis, and during Evodial® mode in: 1 dialysis and in 0 dialyses.

An ANOVA analysis showed that the total dose of heparin (tinzaparin) added during haemodialysis was significantly less (*p* < 0.001) for all other modes than for standard haemodialysis. Clotting tendency resulted in bolus doses of heparin (500–1000 Units) during haemodialysis (Table 1). The need of additional heparin was less for HAC vs. H (*p* = 0.011) and for Evodial vs. H (*p* = 0.031) The added heparin dose was also less for HAC vs. HA (*p* = 0.022). No other significant difference was present. There was no additional dose of heparin/tinzaparin needed in any of the SHD dialyses.

There was no excess in bleeding tendency (such as at the puncture site) during or after haemodialysis for any of the modes. One normally stable participant suffered a serious hypersensitivity reaction that entailed hypotension, nausea and dizziness after 100 min of treatment with Evodial®.

### Efficacy

**Fig. 1:**
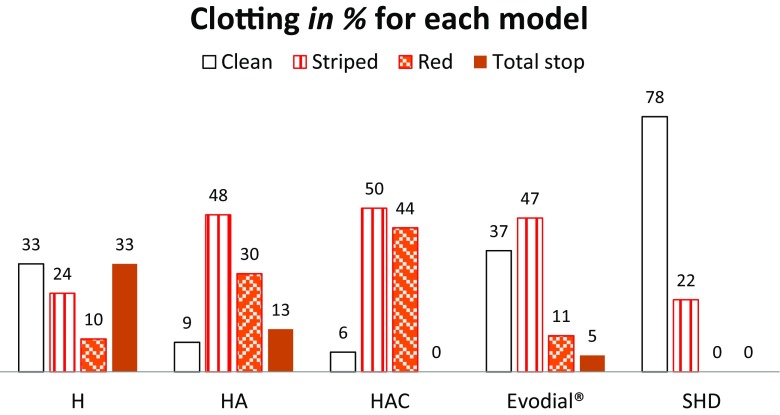
Grades of clotting of dialyzers with the different priming modes and baseline treatment (SHD). H heparin, HA heparin-albumin, HAC heparin-albumin and a citrate-containing dialysis fluid, SHD standard treatment with tinzaparin as anticoagulant

Clotting data for dialyzers and chambers were expressed as the percentage of participants that showed a particular grade of clotting after each mode. These values were pairwise compared using the Wilcoxon rank test. The visually graded clotting of dialyzers and chambers showed significantly less clotting after SHD vs. all other modes (*p* < 0.01). Evodial® had less extensive clotting of dialyzers than HA and HAC (*p* < 0.01). There was no difference in extents of clotting in the chambers between the low-dose modes. The extent of the grades of clotting is shown in Fig. 1.

A total of 11 dialyses (8 individuals, 5 males) were prematurely interrupted (due to ‘total stop’ by clotting) after a mean of 118 min (range 0–218 min). These represented 33% of H, 15% of HA, 5% of Evodial® and none of HAC. Mode H had more interrupted dialyses than SHD (*p* = 0.02) and HAC (*p* = 0.03). H priming resulted in more interrupted dialyses than HA, HAC and Evodial® taken together (*p* = 0.04, RR 3.7, CI 1.2–12).

There was no relation between clotting and access type or between clotting and the kind of catheter lock solution.

Urea reduction rates (URR) at 30 and at 180 min were less effective with Evodial® than with the other modes (*p* < 0.01). In comparing SHD to H (including premature interruptions), a difference in accomplished treatment time was shown (220 ± 23 min vs. 197 ± 64 min, *p* = 0.025) (Table 1).

### Biocompatibility variables

Leukocytes, platelets and lymphocytes were significantly reduced at 30 and 180 min in all modes (*p* ≤ 0.01). There were also changes for the other types of cells (Supplement Table 2). In comparing modes, for the concentration of leukocytes, platelets and lymphocytes at 30 min, leukocytes were more decreased during dialysis with H than with SHD (*p* = 0.012, Wilcoxon). For other differences between modes, see Supplement Table 2. All these values were adjusted for the effect of the ultrafiltration.

### Triglycerides

The median baseline value of triglycerides (TG) was 1.65 (range 0.93–3.98 mmol/L). At 30 min, the median TG levels were reduced with SHD 10% (*p* < 0.001), H by 9% (*p* < 0.01), HA by 11% (*p* < 0.01) and HAC by 9% (*p* = 0.014), but *not* with Evodial® 2% (*p* = 0.34). At 180 min, TG was increased with Evodial® (24%, *p* = 0.010) and HAC (23%, *p* = 0.031).

The reduction of TG at 30 min was most pronounced with SHD compared to H, Evodial® and HAC (*p* < 0.02), while there was no difference compared to HA (*p* = 0.05). Compared to Evodial®, there was a significantly greater reduction of TG with HAC (*p* < 0.05) but not with H or HA. At 180 min, the adjusted increase of TG with Evodial® compared to SHD was the only significant difference between the modes (*p* < 0.05).

Central dialysis catheters with unfractionated heparin as catheter lock solution had a more pronounced reduction of TG at 30 min in comparison to AV fistula (− 13 vs. − 4%, *p* = 0.034) indicating a spillover effect when handling the central dialysis catheter. There was no reduction in TG comparing Taurolock**™**-HEP500 as catheter lock solution for catheters vs. AV fistula (− 6 vs. − 4%, *p* = 0.73).

### Varia

Participants who received warfarin in general received fewer and lower doses of additional heparin than the others (27 sessions, mean 111 ± 253 versus 77 sessions and 344 ± 893 Units/session, *p* = 0.042). There was no difference in heparin administration between participants who were prescribed antiplatelet drugs or not (63/41 sessions 373 ± 975 versus 146 ± 279 Units heparin added/session, *p* = 0.086).

## Discussion

In patients at risk of bleeding, while needing haemodialysis, one option is to use intravenous regional citrate infusion for anticoagulation. This technique is so far only commercially available for continuous veno-venous haemodialysis. Regional citrate anticoagulation requires frequent monitoring and substitution of calcium and careful and frequent surveillance to avoid hyper- or hypocalcaemia [19].Therefore, this technique so far is considered unsuitable for intermittent haemodialysis in untrained hands [8].Therefore, other options may be considered for intermittent haemodialysis in patients at risk of bleeding. The present study investigated four low-dose heparin modes that could be used in such patients. The least need of additional heparin and rise in APTT were found using a priming fluid of saline with heparin and albumin added that was discarded before initiation of dialysis. In addition, subsequent haemodialysis was performed with a citrate-containing dialysate. Similar data were found for the heparin-coated dialyzer Evodial®. Notable, during dialysis withEvodial®, a hypersensitivity reaction occurred in one of the patients in the present study. This had not been reported in previous studies [4, 15]. However, such reaction may develop since the Evodial® dialyzer consists of polyacrylonitrile, a material that can induce such reactions, especially in combination with medications of ACE inhibitors, which were prescribed for the participants in our study. The side effect is induced by bradykinin release [28].

The lower urea reduction rate of Evodial® versus the other modes is most probably due to its lower surface area. The HepZero study reported no change in dialysis efficacy between Evodial® and the control group, using similar surface areas [15].

According to the present study, some patients had increased APTT levels before the start of dialysis. Therefore, initial APTT levels should be established, and if increased, it motivates more precaution when adding any kind of anticoagulant to the participant, and when handling the central dialysis catheters. The finding could be due to insufficient elimination of heparin from the catheter before samples were taken. Another reason could be spillover of heparin from the catheter lock solution into the blood during the preparation and handling of the central dialysis catheter before the start of HD. Such a spillover effect was reported by others when adding heparin as catheter lock solution into the catheter lumen after termination of haemodialysis [23]. The rise in APTT and change in triglycerides indicate that preparing a central dialysis catheter with a heparin-containing lock solution may cause heparin leakage into the blood. This causes a much greater increase of APTT, as marker for bleeding risk, than a small bolus of heparin during the dialysis. This motivates the use of citrate as catheter lock solution in patients at risk of bleeding.

All four low-dose anticoagulant modes in this study contained heparin in some way, including the Evodial® dialyzer with heparin fixed on the membrane surface.

A biological side effect from heparin is that—even by small amounts of heparin—lipoprotein lipase (LPL) is released from its binding sites on the vessel surface. When the LPL is released, a decrease in triglycerides (TG) occurs [22]. Such a significant decrease in TG was found with all modes except Evodial®. This indicates a leakage of heparin from surfaces during haemodialysis with the standard setting and with modes H, HA and HAC, while such leakage does not seem to occur, or is negligible, with Evodial®. Evodial® instead showed a significant *increase* of TG at 180 min. If this by any reason is due to a reduced breakdown of lipids needs to be further investigated. In general, increased TG increase viscosity and thereby increase clotting [24, 25].

Notably, many postoperative patients receive a daily prophylaxis of LMWH despite the presence of, or an imminent risk of, a bleeding. Therefore, if intermittent HD is necessary, a low-dose anticoagulation method, and low doses of heparin boluses added, as mentioned in this study, may be a therapeutic option if the risk of bleeding is considered low. If a severe bleeding is in progress, dialysis should be performed without addition of heparin bolus.

When pulse doses are added, it should be noted that low doses of heparin have shorter half-life than high doses [26]. This motivates the use of small repeated bolus doses of heparin instead of single larger doses to prevent interruption of haemodialysis.

Further evidence of pathophysiological different reactions based on anticoagulation are the different decreases in leukocyte and platelet counts in our study. These are markers for the inflammatory interaction induced between the dialyzer and the blood [27]. The H mode was coupled with the most pronounced reduction in leukocytes as an indicator for more blood membrane interaction. This reaction could also be a reason for the presence of most premature clotting episodes with this mode. There were significant differences also between the anticoagulation modes for monocytes, basophils and eosinophils indicating the activation of these cells also is depending on the dose of heparin but also presence of citrate in the dialysate.

### Limitations

The patients in the present study did not have any bleeding risks, and therefore, the extent of anticoagulation they used may be higher than expected for patients at risk of bleeding. In contrast, patients with multi-organ failure or sepsis, the coagulation system may be affected and the results from the present study may not be immediately transferrable to such patients. In such patients, conditions change over time making a comparative study difficult. Therefore, all treatments were performed on stable participants with chronic kidney failure and a need of regular intermittent dialysis. No participant had an increased bleeding risk. To make sure that the participants received their intended extent of dialysis, the researchers decided not to let the treatment advance to the point of interruption, if this was possible to avoid. The balance between not interrupting their regular treatment cycle and not adding heparin too soon was challenging. There were 20 different nurses performing the treatments, and although they were all experienced, we cannot be sure that the evaluation of the need for extra heparin doses were made equally. The study was open-labelled due to safety, technical and logistical reasons. One also must remember that participants with ongoing bleeding, low platelets or low haemoglobin are more likely to manage a dialysis treatment without any added anticoagulant [29], while the participants in our study were stable chronic end-stage renal disease dialysis participants.

## Conclusion

This study included four low-dose heparin modes that could be used for patients needing intermittent HD while having an increased risk for bleeding. The least need of additional heparin and change in APTT were found using HAC and Evodial®. UsingEvodial® causedone hypersensitivity reaction. Preparing a central dialysis catheter with a heparin-containing lock solution may cause a much greater increase of APTT than a small bolus of heparin during the dialysis.

## Electronic supplementary material

ESM 1 (DOC 63 kb)
