# Supplementary material for: An evaluation of four modes of low-dose anticoagulation during intermittent haemodialysis
Source: Eur J Clin Pharmacol. 2017 Dec 2;74(3):267–74. doi: 10.1007/s00228-017-2389-x (PMC5808085; doi:10.1007/s00228-017-2389-x)
Supplement: Supplementary file 1 — (DOC 63 kb) [file 228_2017_2389_MOESM1_ESM.doc]

**Supplement Table 1:** **Study design,** showing the treatment modes, including baseline standard haemodialysis (SHD), during the two steps regarding priming solution, dialyser and dialysate. All participants received one treatment with each mode in both steps within the study. Differences in number of participants are due to drop out.

|  | **Study model abbreviation** | **Priming solution concentration** | **Dialyser**  **Area**  **Material** | **Dialysate** |
| --- | --- | --- | --- | --- |
|  | **H (n=20)** | Heparin (5000 IU/L)  Saline (0.9 mg/ml) | FX 80  1.8 m^2^  Helixone® | Smartbag® 211.25  or  SmartBag® 311.25 |
| **Step 1** | **HA (n=21)** | Heparin (5000 IU/L)  Albumin (1 g/L)  Saline (0.9 mg/ml) | FX 80  1.8 m^2^  Helixone® | Smartbag® 211.25  or  SmartBag® 311.25 |
|  | ***SHD* (n=23)***  *(Baseline)* | *Saline (0.9 mg/ml)* | *FX 80*  *1.8 m^2^*  *Helixone®* | *Smartbag® 211.25*  *or*  *SmartBag® 311.25* |
| **Step 2** | **HAC (n=19)** | Heparin (5000 IU/L)  Albumin (1 g/L)  Saline (0.9 mg/ml) | FX 80  1.8 m^2^  Helixone® | SelectBag® CX265G Citrate |
|  | **Evodial® (n=19)** | Saline (0.9 mg/ml) | Evodial®  1.6 m^2^  HeprAN | Smartbag® 211.25  or  SmartBag® 311.25 |

H=Heparin priming, HA=Heparin-Albumin priming, SHD=standard haemodialysis, HAC= HA-priming and a citrate containing dialysate.

**Supplement Table 2:** Paired analyses of mean numeric difference expressed as mean difference in percentage (±standard deviation) between start values (0 minutes) versus values (adjusted for the effect of ultrafiltration) at 30 minutes, and the start value versus that at 180 minutes. Wilcoxon signed rank test was used for paired comparisons. Significant differences between start and 30 versus 180 minutes with the same mode are given as ^a)^ (p<0.05) or ^b)^ (p≤0.01). Significant differences between other modes compared to standard dialysis (SHD) as reference are given as ^c)^ (p<0.05); other modes compared to mode H as ^d)^ (p<0.05); other modes compared to mode HA as ^e)^ (p<0.05); HAC compared to Evodial® as ^f)^ (p<0.05).

|  | **SHD** | | **Heparin** | | **HA** | | **Evodial** | | **HAC** | |
| --- | --- | --- | --- | --- | --- | --- | --- | --- | --- | --- |
| Change in % from | Mean | (SD) | Mean | (SD) | Mean | (SD) | Mean | (SD) | Mean | (SD) |
| **0 to 30 minutes:** |  |  |  |  |  |  |  |  |  |  |
| Leukocytes | -7^b^ | (9) | -12^b,c^ | (18) | -11^b^ | (12) | -8^b^ | (9) | -8^b^ | (9) |
| Platelets | -8^b^ | (7) | -5^b^ | (13) | -7^b^ | (8) | -6^b^ | (3) | -7^b^ | (7) |
| Lymhocytes | -16^b^ | (15) | -27^b^ | (20) | -18^b^ | (17) | -20^b^ | (14) | -22^b^ | (14) |
| Monocytes | -14^b^ | (22) | -28^b,c^ | (24) | -23^b^ | (29) | -8^e^ | (20) | -21^b,f^ | (30) |
| Basophils | -1 | (34) | -13^c^ | (42) | -8 | (33) | -3^d^ | (21) | -21^d^ | (51) |
| Eosinophils | 0 | (63) | -25^b^ | (32) | -18^b^ | (58) | -12^e^ | (15) | -3^d,e^ | (22) |
| Neutrophils | -5 | (9) | -6 | (18) | -7^a^ | (13) | -6^b^ | (10) | -3 | (12) |
| **0 to 180 minutes:** |  |  |  |  |  |  |  |  |  |  |
| Leukocytes | -9^b^ | (11) | -8^a^ | (15) | -13^b^ | (13) | -12^b^ | (14) | -10^b^ | (14) |
| Platelets | -6^b^ | (7) | -6^a^ | (14) | -7^b^ | (5) | -6^b^ | (4) | -7^b^ | (5) |
| Lymhocytes | -17^b^ | (21) | -30^b^ | (18) | -24^b^ | (21) | -26^b^ | (21) | -33^b^ | (24) |
| Monocytes | -20^b^ | (24) | -18^a^ | (29) | -27^b^ | (39) | -28^b^ | (30) | -21^b^ | (25) |
| Basophils | -16^b^ | (23) | -19^a^ | (37) | -29^b^ | (38) | -10^a^ | (27) | -23^a,f^ | (51) |
| Eosinophils | -19^b^ | (51) | -39^b^ | (43) | -40^b,c^ | (33) | -26^b^ | (32) | -27^b,d,e^ | (40) |
| Neutrophils | -6 | (16) | 1 | (18) | -4 | (18) | -4 | (20) | 0 | (21) |
